# Mechanisms of Basement Membrane Micro-Perforation during Cancer Cell Invasion into a 3D Collagen Gel

**DOI:** 10.3390/gels8090567

**Published:** 2022-09-07

**Authors:** Shayan S. Nazari, Andrew D. Doyle, Kenneth M. Yamada

**Affiliations:** Cell Biology Section, National Institute of Dental and Craniofacial Research, National Institutes of Health, Bethesda, MD 20892, USA

**Keywords:** hydrogel, 3D culture, imaging, cancer invasion, cell-matrix interactions, proteases, matrix metalloproteinases, actin polymerization, contractility

## Abstract

Cancer invasion through basement membranes represents the initial step of tumor dissemination and metastasis. However, little is known about how human cancer cells breach basement membranes. Here, we used a three-dimensional in vitro invasion model consisting of cancer spheroids encapsulated by a basement membrane and embedded in 3D collagen gels to visualize the early events of cancer invasion by confocal microscopy and live-cell imaging. Human breast cancer cells generated large numbers of basement membrane perforations, or holes, of varying sizes that expanded over time during cell invasion. We used a wide variety of small molecule inhibitors to probe the mechanisms of basement membrane perforation and hole expansion. Protease inhibitor treatment (BB94), led to a 63% decrease in perforation size. After myosin II inhibition (blebbistatin), the basement membrane perforation area decreased by only 15%. These treatments produced correspondingly decreased cellular breaching events. Interestingly, inhibition of actin polymerization dramatically decreased basement membrane perforation by 80% and blocked invasion. Our findings suggest that human cancer cells can primarily use proteolysis and actin polymerization to perforate the BM and to expand perforations for basement membrane breaching with a relatively small contribution from myosin II contractility.

## 1. Introduction

The vast majority of cancer-associated deaths (about 90% of all cases) are caused by metastatic disease rather than primary tumors [1]. During epithelial cancer progression, epithelial cells lose their apical–basal polarity and become less organized, but they remain confined within a basement membrane (BM) barrier at the early cancer stage termed in situ carcinoma. At the second stage of tumor progression, cells penetrate through the basement membrane and invade the surrounding tissue. Cancer cells can then metastasize; they disseminate and migrate away from the primary tumor through the extracellular matrix (ECM), intravasate into blood vessels or the lymphatic system, and then extravasate and form secondary tumors at distant sites. Tumor metastasis is the leading cause of cancer-associated deaths; however, for carcinomas to metastasize they must first break through basement membrane barriers.

Basement membrane (BM) penetration is consequently the first important step in cancer invasion, ultimately leading to tumor metastasis and decreased patient survival. BM degradation also results in the release and activation of growth factors involved in angiogenesis, tumor invasion, and metastasis. Thus, it is important to identify the mechanisms involved in cancer cell breaching of the BM. BMs are thin, sheet-like structures comprising networks of laminin, collagen IV, perlecan, nidogen, and proteoglycans. Laminin can directly bind to cell surface receptors, such as β1 integrin and dystroglycan, to self-assemble into a flat, dense sheet. Collagen IV then polymerizes to form a second covalently crosslinked network. These collagen IV polymers associate with the laminin polymers via nidogen/entactin crosslinks [2,3,4].

The BM serves as a structural layer that encapsulates epithelial and endothelial cells [3,4,5]. The BM is also a nanoporous structure. These submicron pores restrict cell movement and diffusion of large molecules through the BM, while allowing for permeability of small molecules. The size of BM pores varies depending on the tissue type, with the average pore size (the largest distance between fibers in three-dimensional space) of mammary epithelial BM measured to be only ~10 nm [3,6]. During invasion, cancer cells penetrate the BM and migrate within the ECM toward the circulatory or the lymphatic system. Because a cell’s nucleus is the largest organelle with a cross-sectional area ~10 µm^2^, cells must perforate the BM and expand these perforations sufficiently to allow the nucleus and cell body to traverse the BM and invade the surrounding microenvironment [3].

A well-known mechanism cells use to invade through the BM is local proteolysis of the BM and the surrounding stromal ECM. Localized basement membrane degradation requires membrane metalloproteases (MMPs), which also include the family of ADAMs (a disintegrin and metalloproteinase). MMPs are a family of zinc endopeptidases that cleave ECM molecules. ADAMs are enzymes that cleave growth factors, cytokine receptors, and adhesion molecules. MMPs are particularly important enzymes for ECM remodeling during wound healing, development, mammary gland involution, and angiogenesis. Cancer cells can form mechanosensitive actin-based protrusions called invadopodia that locally deliver proteases for ECM degradation and thereby enable penetration through the basement membrane [7,8,9,10]. A classic concept of BM invasion has proposed that cancer cells break through the BM barrier by chemical degradation using these proteases, particularly MMPs.

However, emerging evidence shows that besides proteases, physical mechanisms can also be involved in cell invasion. For example, numerous clinical trials treating patients with broad or more-specific MMP inhibitors failed to reduce mortality. Although this failure could have resulted from inadequate drug dosing or disease states too advanced to respond, recent findings suggest that cells may also be able to breach the BM barrier through physical mechanisms independently of proteases [3,11,12,13]. In a *Caenorhabditis elegans* (*C. elegans*) developmental model of BM invasion, cells can gradually breach the BM without proteases through force application to physically displace the BM, expanding the hole to permit slower but eventual invasion [5]. However, whether human cancer cells can similarly disrupt intact BMs without using any protease activity is not yet known.

Some of the main limitations for studying early events in cell invasion and BM breaching is that invasion is rare and occurs deep in the body, so being able to catch tumors undergoing the initial steps of cancer invasion is challenging. Moreover, tumors excised from humans or animals often lack a continuous BM [14], making it difficult to study the initial cell–ECM interactions at the BM interface. In contrast, we previously created an in vitro model of invasion that uses confocal microscopy to visualize human cancer cells as they perforate the BM for studies of the mechanisms of BM perforation and hole expansion during cancer invasion [15].

Recent studies on BM breaching have tended to focus on individual cells in 3D collagen gels, *C. elegans* models, or synthetic 3D cell cultures to characterize invasion and migration. Previous studies have also examined the contributions of myoepithelial, stromal, and immune cells to BM perforation and cancer cell invasion [16,17,18,19]. Here, in this study, we focused on the contributions of cancer cells themselves to BM penetration and invasion with an emphasis on establishing the cellular mechanisms. We report that during early human cancer cell invasion from cancer spheroids, the BM was initially perforated by cells using actin polymerization, protease degradation, and a modest contribution from actomyosin contractility.

## 2. Results and Discussion

### 2.1. Overview of the 3D-Spheroid Model

**Scheme 1:**
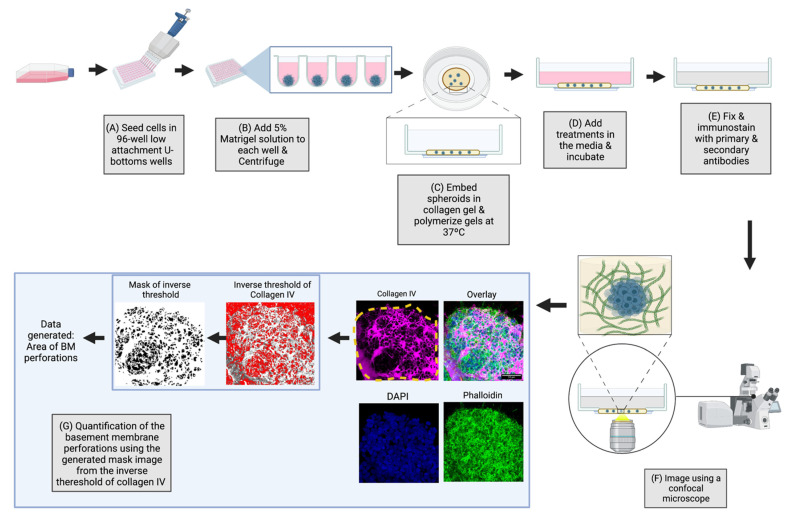
Schematic illustration of the protocol for generating cancer cell spheroids encapsulated in a basement membrane and embedded in a 3D collagen gel for invasion assays. (**A**) In the spheroid generation process, cells were seeded in a 96-well, low attachment V- or U-bottom dish. To form aggregates, after 8 h the plate was centrifuged at 300 RPM for 5 min. (**B**) To form spheroids with an intact basement membrane, a dilute solution of Matrigel was added to the spheroids at a final concentration of 5% Matrigel per well. The plate was centrifuged again at 300 RPM for 5 min. (**C**) After the plate had incubated for several days at 37 °C, the spheroids were embedded in neutralized collagen gels at 4 mg/mL gel concentration, and the gel was polymerized at 37 °C for 1 h. (**D**) Culture medium and any treatments (if applicable) were added to the spheroids in the dishes. (**E**) At the appropriate time, spheroids were then fixed with 4% paraformaldehyde and immunostained with primary (collagen IV) and secondary antibodies accompanied by DAPI (nuclear stain) and phalloidin (F-actin stain). (**F**) Spheroids were imaged via confocal microscopy. (**G**) For basement membrane perforation quantification, maximum intensity projections of the collagen IV channel were generated, and a region of interest was drawn around the boundary of the BM to delineate the total spheroid area (yellow dashed line). From this ROI, we generated an inverse threshold image and subsequently a masked version of it, from which we calculated the area of each perforation within the ROI, as well as the area of the ROI.

**Figure 1:**
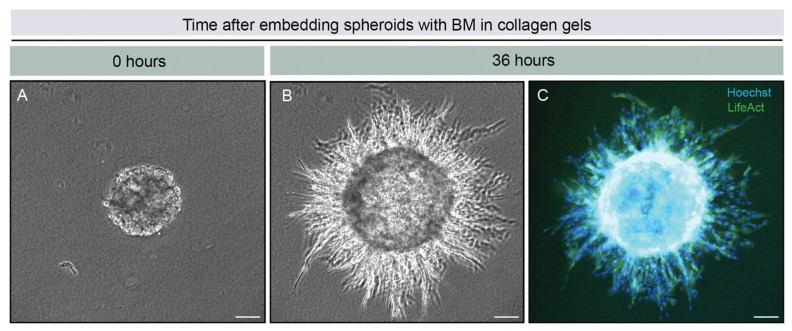
Spheroid invasion through the basement membrane and into a collagen gel. (**A**) Spheroid after embedding in a collagen gel and polymerizing the gel (time 0 h) compared to a spheroid after 36 h (**B**,**C**). Cancer cells in the spheroid invaded into the surrounding collagen gel and exhibited a “sunburst” phenotype which entails a trail of cells outward from the main spheroid body. We noted that, besides the outward invasion of cancer cells into the collagen gel, the spheroid expanded at 36 h due to ongoing cell proliferation. Scale bars: (**A**–**C**), 100 µm.

To investigate the mechanisms of human cancer cell breaching through the BM, we used our three-dimensional in vitro invasion model (Scheme 1). The spheroids were first encapsulated within a BM, and then the BM-containing spheroids were embedded in 3D collagen gels. Over the next ~18 to 36 h, the cells migrated from the spheroid and into the collagen microenvironment through collective cell migration, often in a “sunray” or “sunburst” pattern comprising narrow columns of cells (Figure 1).

### 2.2. BM Encapsulated Spheroids before Invasion and Became Extensively Perforated in Our Three-Dimensional In Vitro Invasion Assay

**Figure 2:**
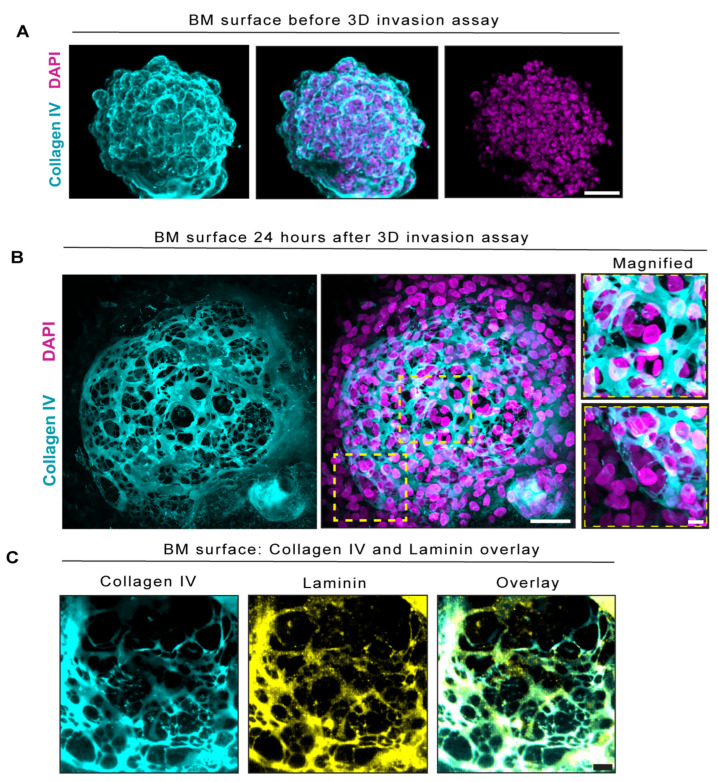
Large perforations in the basement membrane appeared as cancer cells invaded. (**A**) Images show a spheroid with a basement membrane visualized by immunostaining for anti-collagen IV antibody and DAPI (staining for nuclei) before it was embedded in collagen gel. We observed no apparent holes in the basement membrane before the invasion assay. (**B**) 24 h after the spheroids were embedded in collagen gels, large holes were visible with collagen IV immunostaining (cyan) of the basement membrane (upper inset). DAPI staining denoting cell nuclei (magenta) demonstrated many cells outside of the basement membrane (lower inset). (**C**) Immunolocalization of collagen IV (cyan) and laminin (yellow) staining in the in vitro basement membrane. These figures are representatives of at least 3 independent experiments, each including at least 3 spheroids per experiment, all of which showed similar results. Scale bars: (**A**,**B**), 100 µm; Inset of (**B**,**C**), 10 µm.

We first evaluated whether any microscopic holes existed in the BM prior to invasion by fixing spheroids prior to embedding in collagen I gels that were 20 mm in diameter and approximately 500 µm thick. As we have shown previously [15], immunostaining and confocal microscopy for collagen IV revealed a relatively continuous sheet of varying thickness (approximately 0.2–5 µm thick) surrounding the spheroid (including the peripheral cells of the spheroid) with few apparent holes (Figure 2A). After 24 h incubation in culture media, followed by fixing and immunostaining for collagen IV, we observed perforations in the BM with varying sizes ranging from submicron to holes that could fit multiple nuclei (Figure 2B—magnified images). As predicted, we also observed many nuclei outside of the perforated BM suggesting that many cells invaded through the BM. The observation of holes larger than the diameter of a nucleus (the largest organelle of a cell) contrasted with previous characterizations of non-cancerous embryonic tissues by our laboratory in which micro-perforated BM surrounded expanding embryonic epithelia of lung, kidney, and salivary glands during branching morphogenesis [20]. These microscopic holes in embryonic BM were smaller than the diameter of the underlying epithelial cells and only contained the cytoplasmic extensions of the epithelial cells that protruded through these holes, but the cell bodies did not cross the BM. Recently, another laboratory observed similar micro-perforations in the BM that encases the embryo during mouse embryogenesis [21]. In contrast with the perforations during embryonic branching morphogenesis and mouse embryogenesis, the assay with cancer spheroids revealed cells within larger BM perforations with many cells that penetrated through the BM and traversed into the collagen gel (Figure 2B). In the magnified images of Figure 2B we observed some holes containing one or more cells traversing the BM. The collective cell migration mode, showing the “sunray” phenotype of radially oriented columns of invading cells, demonstrated that although cells could perforate and traverse many holes in the BM, they often trailed behind leader cells in this collagen gel, which may have provided a path of least resistance. Laminin also was present and co-immunolocalized with collagen IV staining in the BM (Figure 2C). Consequently, although the source of BM we used in this assay was a diluted, soluble basement membrane extract (Matrigel), the spheroid cells could assemble overlapping the collagen IV and laminin networks that fully encapsulated the spheroid before any subsequent formation of perforations and invasion.

### 2.3. BM Perforations Expanded over Time as Cancer Cells Initiated Invasion through the BM

**Figure 3:**
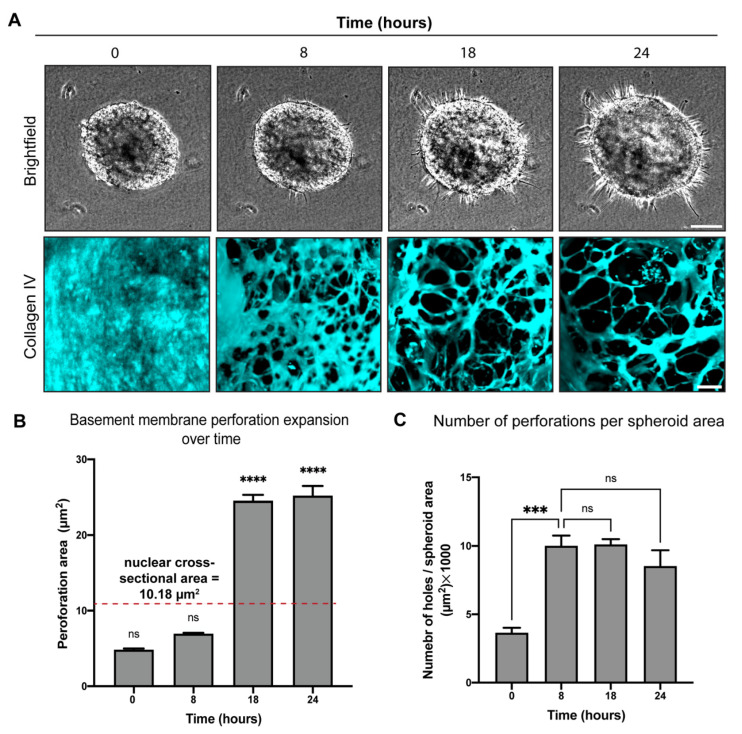
Basement membrane perforation over time. (**A**) Brightfield images of a spheroid from a time-lapse video spanning 24 h (**upper images**) and basement membrane perforations from the same time points depicted by collagen IV staining (**lower images**). (**B**) Quantification of the average areas of individual basement membrane holes (±SEM) at each of the corresponding time points (**lower images**). Dashed line on the graph indicates the mean nuclear cross-sectional area of MDA-MB-231BO cancer cells. (**C**) Quantification of the number of holes per spheroid area. **** *p* < 0.0001 *** *p* < 0.0003. These graphs are based on pooled data from at least 3 independent experiments each including at least 3 spheroids per experiment, all of which showed similar results. Scale bars: (**A**), **top row**, 100 µm; (**A**), **bottom row**, 10 µm.

To examine whether the BM perforations expand over time and whether their expansion correlates with cell invasion through the BM, we quantified the areas of the individual perforations in the BM over time (Figure 3B). To understand the progression of the BM perforation process, we embedded spheroids in a collagen I gel, and after polymerization (1 h at 37 °C), fixed samples at 0 (1 h after polymerization), 8, 18, and 24 h. Small perforations in the BM appeared by time 0 after gel polymerization (Figure 3A—time 0-h). By 8 h after spheroid embedding in collagen gels, the BM showed a significant increase in the hole number (~2.7-fold increase), yet with perforation areas similar to the 0 h timepoint, but with no visible invasion across the BM (Figure 3B,C). The absence of cellular invasion at 8 h (mean perforation area: 6.9 µm^2^ ± 8.1 SD) was consistent with the finding that the average nuclear cross-sectional area was 10.2 µm^2^ ± 0.3 SD, i.e., often too large to pass through the perforation at 8 h, which thus likely prevented effective cell invasion through the BM (Figure 3B). Invasion was evident at 18 h when the perforation size exceeded the nuclear cross-sectional area (Figure 3A,B). However, the perforation number did not change between 8 h (pre-invasion) to 18 h (after invasion), suggesting that the subcellular holes were not simply merging to make larger holes but rather expanding over time (Figure 3C). Interestingly at 24 h, while more cells were observed trailing behind the leader cells breaching the BM and invading further out into the 3D collagen gel, there was no change in the hole size or number (Figure 3C), suggesting that the cells did not need to generate more or expand existing holes and simply followed along paths of least resistance.

### 2.4. Protease Activity Played a More Important Role Than Actomyosin Contractility in Perforating the BM during Cancer Cell Invasion

Developmental models of invasion have revealed that besides proteases, other mechanisms can play a role in the formation and expansion of perforations [3,20]. One mechanism is actomyosin contractility [20]. Previously, our laboratory had discovered that during embryonic salivary gland branching morphogenesis, epithelial expansion depends on both proteases for BM distensibility and perforation and actomyosin contractility for expanding BM perforations and remodeling the matrix, with these two processes apparently synergizing [20].

**Figure 4:**
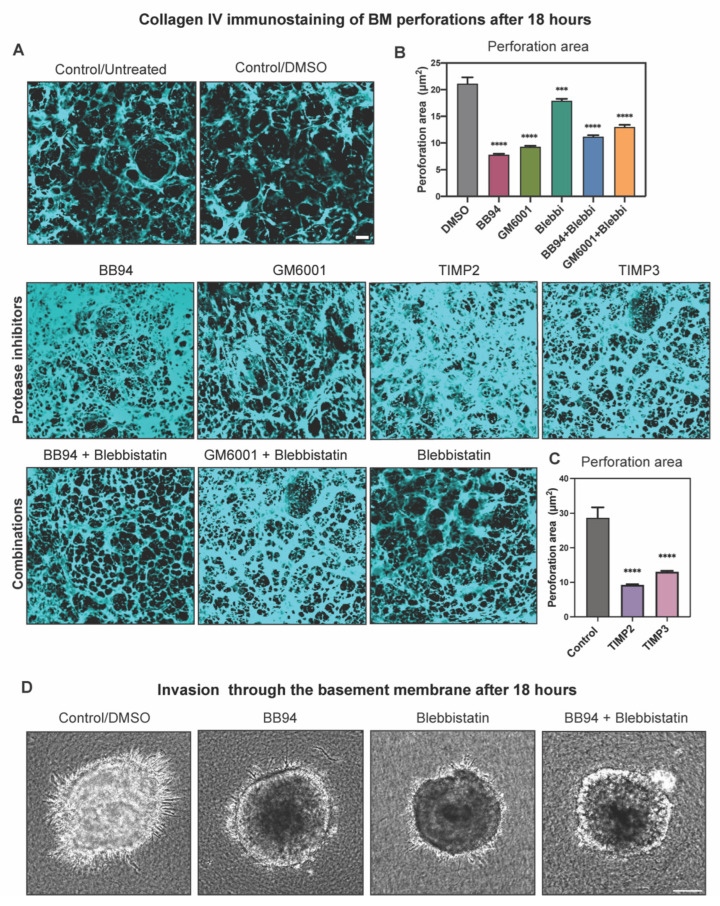
Contributions of proteolysis and myosin II contractility to basement membrane perforation. (**A**) Maximum intensity projection images of the basement membrane surrounding the spheroid after 18 h treatment with the following protease inhibitors or combinations of inhibitors: control (untreated), control (DMSO), BB94, GM6001, TIMP2, TIMP3, blebbistatin, BB94 + blebbistatin, or GM6001 + blebbistatin. (**B**) Quantification of basement membrane perforation areas after treatment with the indicated small molecule inhibitors during 18 h cancer cell invasion. (**C**) Quantification of perforation area in comparisons of TIMP2 and TIMP3 versus control. (**D**) Brightfield images of spheroids shown in panel A treated with small-molecule inhibitors of proteases (BB94), myosin II (blebbistatin), or a combination of BB94 and blebbistatin. **** *p* < 0.0001; *** *p* < 0.0007. These graphs and images are based on pooled data from at least 3 independent experiments, each including at least 3 spheroids per experiment, all of which showed similar results. Scale bars: (**A**), 10 µm; (**D**), 100 µm.

To determine the mechanisms of perforation expansion by cancer spheroids, we first used small molecule inhibitors to disrupt proteolysis and actomyosin contractility. Because the mean perforation cross-sectional area exceeded the nuclear cross-sectional area of cells at 18 h, we chose this time point for assaying the inhibitor treatments. After treating spheroids with a variety of MMP protease inhibitors (BB94, GM6001, TIMP2, and TIMP3) for 18 h, we observed substantial reductions in the size of the holes accompanied by increased fluorescence intensity of collagen IV immunostaining, suggesting an apparent thickening (inhibition of turnover; data not shown) of the BM (Figure 4A,B). This suppression of perforation expansion was accompanied by decreased invasion of cancer cells (Figure 4D). Specifically, batimastat (BB94) produced one of the most dramatic reductions in the sizes of the holes with a 63% reduction compared to control, while GM6001, a less broad-range inhibitor, still substantially inhibited perforation size by 56% (Figure 4B) and significantly reduced cell invasion (Figure 4D). The natural protease inhibitor TIMP2 (tissue inhibitor of metalloproteinases-2) that inhibits a subset of MMPs had the most dramatic effect on the perforation cross-sectional area, reducing it by 68% and inhibiting cell invasion compared to its control (Figure 4C). TIMP3, which inhibits several MMPs and ADAMs (a disintegrin and metalloprotease), had a lower (55% reduction) but significant effect on inhibition of the perforation area (Figure 4C). Inhibition of actomyosin contractility with blebbistatin (a specific myosin II ATPase inhibitor) had modest effects on the BM perforation areas (15% reduction) compared to the major effects of protease inhibition (Figure 4B). These results are consistent with numerous previous studies implicating proteases in BM breaching. However, while contractility has been reported to aid in perforating the BM during breaching, our findings indicate that proteolysis, and not actomyosin contractility, appeared to be most important for perforation expansion to permit cancer cells to invade in this human spheroid system.

To test for potential cooperativity between proteolysis and actomyosin contractility during hole formation by spheroid cancer cells, we treated the spheroids with a combination of BB94 and blebbistatin or with GM6001 plus blebbistatin, which reduced the perforation area by 47% and 38%, respectively, compared to their controls (Figure 4A—bottom row and Figure 4B). Although these combined treatments resulted in significant reductions in perforation size and cell invasion, in both cases, this reduction was not quite as dramatic as even BB94 or GM6001 alone. That is, there was clearly no evidence for cooperation between proteolysis and actomyosin contractility in this cancer cell spheroid invasion model. These findings contrast with the results in a developmental model of BM perforation where treatment of embryonic salivary glands [20] with similar inhibitor combinations had a more rapid and dramatic effect on BM perforation than either treatment alone. In a *C. elegans* model for invasion, actomyosin contractility could even eventually drive BM breaching in the absence of protease activity [5]. Our results, differing from developmental models, suggested that there could be other mechanisms contributing to hole formation in the BM during the invasion of cancer cells.

### 2.5. Actin Polymerization Dramatically Affected Hole Formation and Acted as a Third Mechanism Contributing to Perforation of the BM

**Figure 5:**
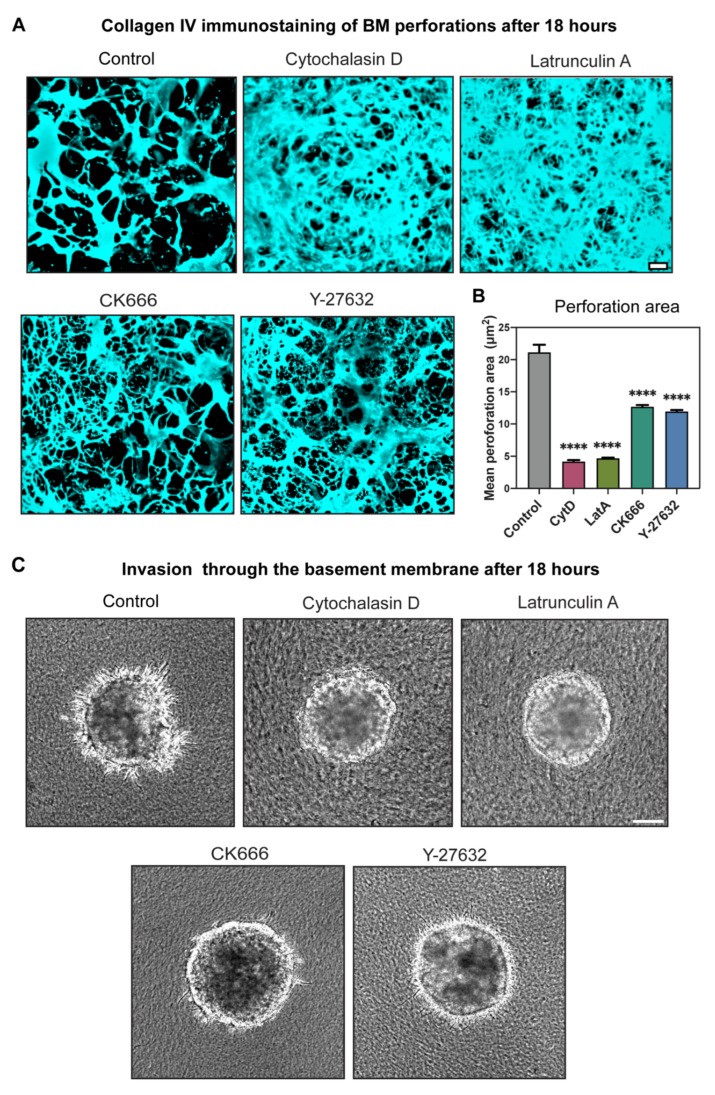
Contribution of actin polymerization to basement membrane perforation during collective cancer cell invasion. (**A**) Confocal immunofluorescence images of the basement membrane (anti-collagen IV antibody) after treatment with the following inhibitors: cytochalasin D (actin), latrunculin A (actin), CK-666 (ARP2/3), or Y-27632 (ROCK). (**B**) Quantification of basement membrane perforation areas for each treatment at 18 h. (**C**) Brightfield images of the spheroids after each treatment, showing the effects of the inhibitors on cell invasion. **** *p* < 0.0001. These graphs and images are based on pooled data from at least 3 independent experiments, each including at least 3 spheroids per experiment, all of which showed similar results. Scale bars: (**A**), 10 µm; (**C**), 100 µm.

A classical cytoskeletal mechanism for the formation of cellular protrusions is actin polymerization. In the *C. elegans* model of BM invasion, cells can initially perforate the BM using actin-based invadopodia and actomyosin contractility with a subsequent large cellular protrusion to penetrate through the BM without the use of proteases by applying force to deform the BM, thereby enabling a slower but eventual invasion [3,5]. However, a role for actin polymerization in collective cancer cell invasion through a BM has not been fully explored. To test for a role for this mechanism, we inhibited actin polymerization using either cytochalasin D or latrunculin A. Both small molecule inhibitors had dramatic inhibitory effects on hole formation and expansion (Figure 5A,B). To inhibit the F-actin nucleating complex ARP2/3, we used the small molecule inhibitor CK666, which provided evidence that actin branching polymerization played a role in expansion of perforations in the BM (Figure 5A—bottom left panel). Turning to the effects of these inhibitors on the process of invasion, inhibiting actin polymerization and perforation expansion completely inhibited cancer cell invasion through the BM (Figure 5C). The Rho-kinase inhibitor Y-27632 also significantly decreased the perforation size in the BM (Figure 5A,B) and decreased cell invasion (Figure 5C). Y-27632 can inhibit actin polymerization through Diaphanous-related formins (Dia1–3) and cofilin [22,23], which could explain why it inhibited the expansion of perforations more than inhibiting actomyosin contractility using blebbistatin (Figure 4).

### 2.6. Comparisons with Other Cell Types Confirmed Findings for Another Metastatic Cell but Not for a Non-Metastatic Cell Line

We tested a second metastatic cell line to determine whether its spheroids would also generate large BM holes in our in vitro cancer invasion assay. We used 4T1 cells, a murine mammary tumor line that is generally very aggressive. After generating 4T1 spheroids with a BM and embedding them in collagen gels, we observed that, at 24 h, these cancer cells similarly invaded through the BM and out into the surrounding collagen gel (Figure S1A). Additionally, immunofluorescence anti-collagen IV staining revealed that there were large holes in the BM similar to those formed by the MDA-MB-231BO cell line (Figure S1D). We tested several inhibitors for their ability to suppress 4T1 invasion that targeted MMPs, contractility, and actin polymerization and found that they similarly suppressed invasion compared to controls (Figure S1A–C,E,F). Overall, these results confirmed findings with the main cell line we had tested.

To determine whether invasion and BM holes would be generated by a non-metastatic human cell line, we used MCF10A cells in our invasion assay. Their spheroids were similarly encapsulated in a BM and were embedded in collagen gels. After 24 h, however, we found no apparent holes in the BM or outward invasion into the collagen gel (Figure S2A).

In summary, we found that a metastatic cancer line from another species mimicked the BM perforation and outward invasion into collagen gels from spheroids, whereas a non-metastatic human cell line did not. We note that one limitation of this study is that we did not test large numbers of other types of cancer cells to determine whether the mechanisms we described for forming and expanding BM perforations were displayed by all cancers. A second limitation is that these are obviously in vitro model studies, so examining these mechanisms in vivo would be valuable if future technical advances could make it feasible to evaluate the mechanisms of these initial steps of BM breaching by human cancer cells in living tissues.

## 3. Conclusions

Taken together, our results indicate that there were multiple mechanisms contributing to cancer cell BM breaching. They included actin polymerization and proteolysis, with a lesser contribution from actomyosin contractility. It was originally hypothesized that chemical degradation by proteases is key to BM penetration and invasion. From the developmental models, our understanding of invasion expanded to include the contribution of myosin contractility to invasion. The current study focusing on human cancer cell spheroids in a 3D in vitro model of invasion further confirmed that tumor cells could use proteases for initial perforation of the BM. However, we found that the sizes of the perforations were important: when they were smaller than the diameter of a cell, there was minimal invasion, but once the perforation area expanded beyond this size threshold needed to allow cells to traverse the BM, the cancer cells invaded outward. Importantly, we also identified a key role for actin polymerization in order for cell protrusions to expand the BM perforation areas to be able to invade, with a much lower requirement for actomyosin contractility than had been predicted from embryological models. Consequently, the dual functions of MMP-based proteolysis and cytoskeletal actin polymerization were crucial for effective BM perforation and human tumor cell breaching of the BM barrier to mediate cancer invasion.

## 4. Materials and Methods

### 4.1. Cell Culture and Media

We used MDA-MB-231 Bone (MDA-MB-231BO) cells originally described in [24] and obtained from Dr. Kandice Tanner, National Cancer Institute. The culture medium used was Dulbecco’s MEM (DMEM; Gibco) with 10% FBS (Life Technologies), 1% penicillin/streptomycin (Life Technologies), and 1% L-glutamine (Life Technologies). Media were sterile filtered through 0.45 µm nitrocellulose filters. Cells and spheroids were maintained in a humidified 10% CO_2_ incubator at 37 °C.

#### 3-Dimensional Spheroid Cell Culture

Detailed protocols for this method were published previously [15] and are illustrated in Scheme 1. Briefly, MDA-MB-231 BO cells were seeded at a 500 cells-per-well density in ultra-low attachment V-bottom (or U-bottom) 96 well plates (PrimeSurface from s-Bio). After 8 h at 37 °C, the plate was centrifuged for 5 min at 300 RPM and placed back into the tissue culture incubator for 48 h to permit the cells in the spheroid to form a compact spheroid via cell–cell adhesion. We then added a final concentration of Matrigel diluted to 5% in medium per well and centrifuged for 5 min at 300 RPM. The plate was then incubated for at least 48 h further to induce basement membrane assembly around the spheroid. The spheroids were then washed in cold HBSS (Hanks balanced salt solution, Life Technologies) at least 3 times and embedded in 4.0 mg/mL rat-tail collagen I gels. The gels were polymerized at 37 °C for 1 h, and serum-containing cell media or imaging media was added to the dish after the incubation period. We had also tested 2 mg/mL collagen gels, but we ultimately chose to use 4 mg/mL gels because they proved less likely to tear or detach from our MatTek culture dishes during the rigorous washing of our immunostaining protocol.

### 4.2. Inhibitors

For experiments in which spheroids were treated with inhibitors, the spheroids were embedded in neutralized collagen gels and, after polymerization, media were added containing the following treatments: BB94 (5 µM), GM6001 (20 µM), TIMP2 (4 µg/mL), TIMP3 (4 µg/mL), Y-27632 (20 µM), blebbistatin (20 µM), ML141 (20 µM), cytochalasin D (2 µM), or latrunculin A (200 nM). DMSO was used as the vehicle control for the inhibitors that were dissolved in DMSO.

### 4.3. Immunostaining

Spheroids embedded in collagen gels were fixed using 4% paraformaldehyde in PBS for at least 1 h, then washed with PBS and blocked with 10% donkey serum for at least 1 h. Primary antibody against collagen IV antibody (Millipore), goat host, was added to the dish and incubated at 4 °C overnight. After washing the 3D assay with PBS, embedded spheroids were incubated with secondary antibodies (Jackson ImmunoResearch) IgG Fab donkey Cy5 labeled anti-goat for at least 4 h at room temperature before imaging.

### 4.4. Confocal Imaging

Confocal imaging was performed on a Nikon A1R MP + HD confocal system (Nikon Instruments, Melville, NY, USA) using a 40× Apo long working distance (LWD) water immersion objective (N.A. 1.15). Laser lines of 405 nm, 488 nm, 561 nm, and 640 nm provided illumination for Hoechst, AF 488, Rhodamine Red X, and AF647 fluorophores, respectively. Data were acquired using Galvano mode at 1024 × 1024 with no line averaging. A Z-piezo stage (Physik Instrumente USA, Auburn, MA, USA) allowed for rapid imaging in Z every 0.5 µm over a 200 µm Z distance. NIS-Elements (Nikon, Melville, NY, USA) controlled all equipment. All images shown are maximum intensity projections and were processed using ImageJ/FIJI.

### 4.5. Live Cell Imaging

The brightfield live-cell images were obtained using a Nikon Ti-E inverted microscope (Melville, NY, USA) with motorized stage (Prior) using 10× (N.A. 0.3) and 20× (N.A. 0.75) air objectives. Images were acquired with a Hamamatsu Orca Flash 4.0 CMOS camera. NIS-Elements (Nikon, Melville, NY, USA) controlled all equipment. An environmental chamber (Precision Plastics, Beltsville, MD, USA) kept cells at a constant 37 °C, 50% humidity and 10% CO_2_.

### 4.6. Perforation Area Analysis

To semi-automatically quantify the basement membrane hole number and area, a Fiji (ImageJ) macro was created by ADD and can be found at https://github.com/addoyle1D/BM_Holes, (accessed on 9 August 2022). Briefly, a maximum intensity projection (MIP) was defined and created to which an unsharped mask (radius = 15 mask = 0.6) and a Li threshold were applied. A hand-drawn region of interest (ROI) was created over the filtered MIP to include only holes within the center-bottom region of the spheroid, and holes overlapping the edge of the ROI were excluded (see Scheme 1 for an example ROI). The function, “Analyze Particles” was then used to calculate the hole areas and number, excluding any hole that touched the ROI edge or was less than 1.5 microns. All images were then automatically saved as tiff and csv files.

### 4.7. Statistics

We repeated each experiment at least 3 times and each experiment contained at least 3 spheroids. For the perforation area statistical analyses, we used One-way ANOVA with Dunnett’s test.

## Data Availability

Data is available from authors upon request.

## Supplementary Materials

The following supporting information can be downloaded at: https://www.mdpi.com/article/10.3390/gels8090567/s1. Figure S1: 4T1 spheroids showed perforations in the basement membrane and invasion into collagen gel. Figure S2: A non-metastatic cell line did not generate large holes or invade into collagen gels.
