# Supplementary material for: Mechanisms of Basement Membrane Micro-Perforation during Cancer Cell Invasion into a 3D Collagen Gel
Source: Gels. 2022 Sep 7;8(9):567. doi: 10.3390/gels8090567 (PMC9498339; doi:10.3390/gels8090567)
Supplement: Supplementary file 1 [file gels-08-00567-s001.zip › gels-1885547-supplementary.pdf]

Supplementary Materials

Mechanisms of Basement Membrane Micro-Perforation during Cancer Cell Invasion into a 3D Collagen Gel

Shayan S. Nazari, Andrew D. Doyle and Kenneth M. Yamada *

Cell Biology Section, National Institute of Dental and Craniofacial Research, National Institutes of Health, Bethesda, MD 20892, USA

* Correspondence: kenneth.yamada@nih.gov

Figure S1: 4T1 spheroids show perforations in the basement membrane and invasion into collagen gel

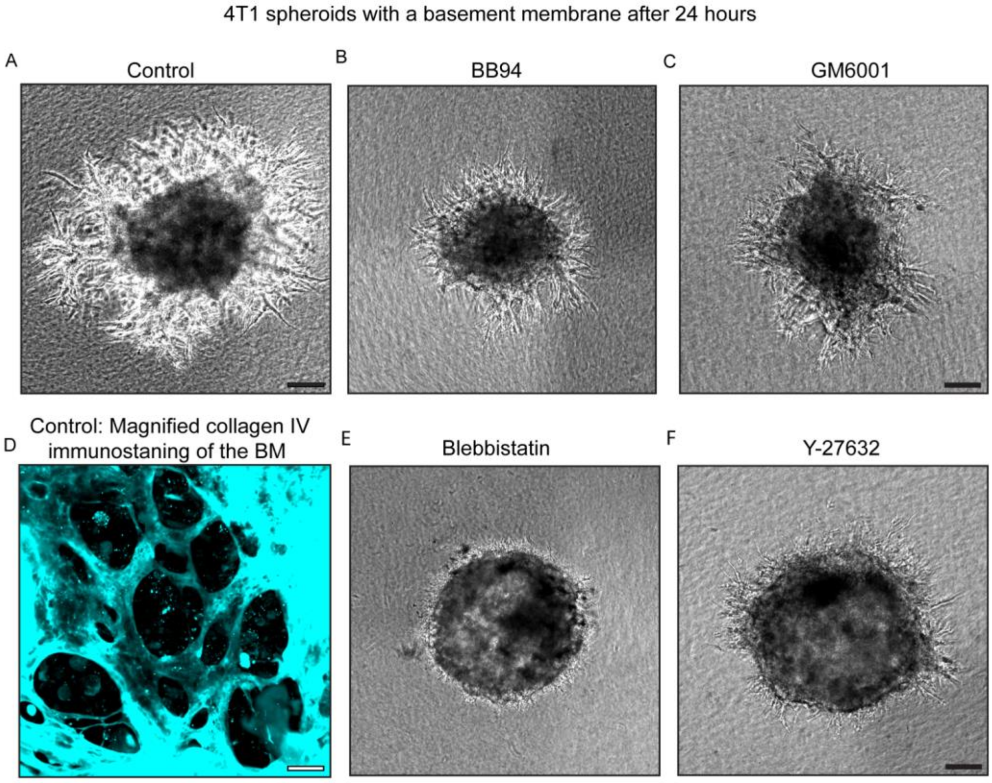

(A) Spheroids from a murine tumor cell line, 4T1, invade into the collagen gel after 24 hours. (B-C) Protease and MMP inhibition with BB94 and GM6001 show decreases in cell invasion similar to those for the MBA-MB-231BO cell line. (D-E) Inhibition of myosin II and ROCK/actin polymerization also suppresses cell invasion compared to control (D-E). Perforations are seen in the basement membrane of 4T1 control spheroids 24 hours after invasion similar to the MDA-MB-231BO cell line. Scale bars: A-C & E-F, 100 μm; D, 20 μm.

Figure S2: A non-metastatic cell line does not generate large holes or invade into collagen gels

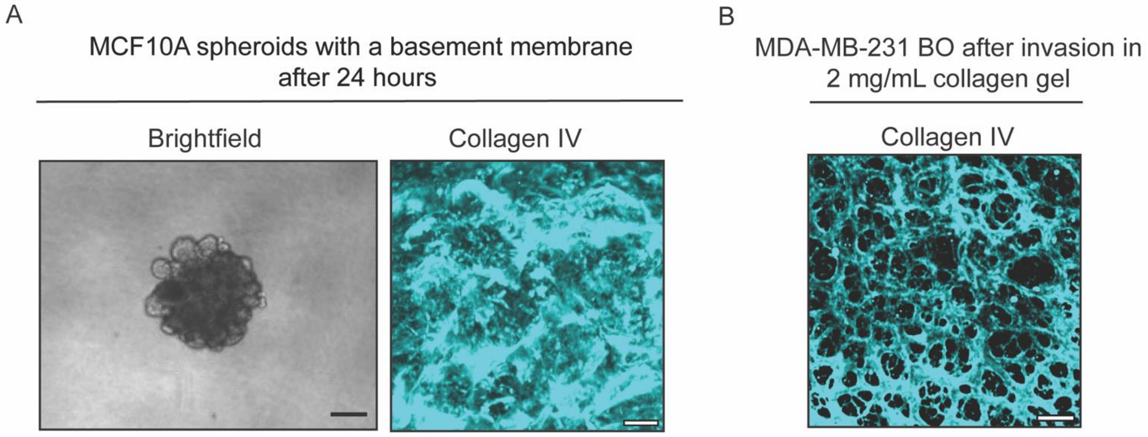

(A) The normal-appearing non-metastatic mammary cell line MCF10A forms a spheroid with a basement membrane and after embedding in collagen gel for 24 hours, the cells do not invade and lack the large holes in the basement membrane. (B) MDA-MB231BO spheroids embedded in 2 mg/mL collagen gels form holes in the basement membrane, similar to MDA-MB-231BO embedded in 4 mg/mL collagen gels. Scale bars: A (left panel), 100 µm; A (right panel), 10 µm; B, 10 µm.
